# Universal COVID-19 screening at hospitals in a large Canadian health region

**DOI:** 10.1017/ash.2023.296

**Published:** 2023-09-29

**Authors:** Matthew Garrod, Katy Short

## Abstract

**Background:** Hospitals were affected by COVID-19, with significant concern regarding transmission from unidentified cases. Fraser Health, a Canadian regional health authority, implemented universal testing along with screening questions for emergency department (ED) admissions. We sought to determine which factors were associated with SARS-CoV-2–positive test on admission as well as patient outcome, stratified by screening question responses. **Methods:** This retrospective analysis included patients aged ≥6 years admitted through 12 hospital EDs between November 1, 2020, and June 30, 2022. Admission, laboratory, and screening data were extracted from electronic health records. Patients who had a first SARS-CoV-2 PCR–positive test in the prior 60 days collected within 48 hours of admission were classified as positive. Covariates included age, geographical region, and SARS-CoV-2 variant era. All questions were modeled using multinomial logistic regression, with components informed through crude analysis in R Studio software. **Results:** There were 88,511 unique eligible admissions, with 7,642 positive tests (8.6%). The positivity rate over the study period ranged from 0.6% to 21.8%, with a mean of 6.5%. Patients meeting screening criteria were 4.7 times (95% CI, 4.43–4.92) as likely to test positive as those who did not. Patients in the SARS-CoV-2 omicron variant era were 3.2 times (95% CI, 2.98–3.47) as likely to test positive as those in the earlier era of the pandemic. Patients later in the pandemic were less likely to be identified by screening questions than those in earlier eras, with patients in the SARS-CoV-2 omicron variant era only 14% (95% CI, 12%–17%) as likely as in the earlier stages of the pandemic to be identified by screening questions. Patients who tested positive were 1.5 (95% CI, 1.37–1.64) times as likely to die as patients who tested negative, whereas patients in later stages of the pandemic were less likely to die overall. **Discussion**: Patients who tested positive on admission were more likely to meet screening criteria; however, screening missed half of all positive cases. It is not known whether patients who tested positive without meeting screening criteria would have resulted in transmission. **Conclusions:** Due to changes in COVID-19 epidemiology, Fraser Health has discontinued universal admission screening. Although universal testing increased resource needs, more than half of patients who tested positive during the study period would not have been identified based on screening criteria alone, allowing for implementation of precaution measures to prevent possible transmission. Ultimately, the decision to conduct universal testing must be a balance of the resources required, community prevalence, and patient population.

**Disclosures:** None

